# Socio-Economic Instability and the Scaling of Energy Use with Population Size

**DOI:** 10.1371/journal.pone.0130547

**Published:** 2015-06-19

**Authors:** John P. DeLong, Oskar Burger

**Affiliations:** 1 School of Biological Sciences, University of Nebraska—Lincoln, Lincoln, Nebraska, United States of America; 2 School of Anthropology and Conservation, University of Kent, Canterbury, United Kingdom; Cinvestav—Merida, MEXICO

## Abstract

The size of the human population is relevant to the development of a sustainable world, yet the forces setting growth or declines in the human population are poorly understood. Generally, population growth rates depend on whether new individuals compete for the same energy (leading to Malthusian or density-dependent growth) or help to generate new energy (leading to exponential and super-exponential growth). It has been hypothesized that exponential and super-exponential growth in humans has resulted from carrying capacity, which is in part determined by energy availability, keeping pace with or exceeding the rate of population growth. We evaluated the relationship between energy use and population size for countries with long records of both and the world as a whole to assess whether energy yields are consistent with the idea of an increasing carrying capacity. We find that on average energy use has indeed kept pace with population size over long time periods. We also show, however, that the energy-population scaling exponent plummets during, and its temporal variability increases preceding, periods of social, political, technological, and environmental change. We suggest that efforts to increase the reliability of future energy yields may be essential for stabilizing both population growth and the global socio-economic system.

## Introduction

Understanding the factors that regulate the size of human populations is crucial to the development of an ecologically sustainable society. Current views about what sets population growth rates in contemporary human societies, however, are poorly integrated and suggest important roles for diverse factors such as water [1], economics [2], development [3], immigration [4], age structure [5], energy [6], evolved fertility behaviors [7], and cultural evolution [8]. This is unfortunate because forecasts of global human population size are still based strictly on statistical extrapolation of historical trends and not on mechanisms of population regulation [6,9], leaving great uncertainty about expectations for future population size [3,10].

One important potential mechanism of population regulation in humans is a negative relationship between population size and per capita resource availability, which generates density-dependent, or ‘Malthusian’, growth [11–15]. (Note that in this paper, for simplicity, we will talk about the ‘size’ of human populations, even though the feedback from population size to population growth rate is known as ‘density’ dependence. Population density is population size per area, and in this paper area is constant through time in all cases except the United States.) Given a constant resource supply to a population, the per capita availability of resources declines as the population grows. As resources become scarce, individuals consume less, driving down birth rates and/or raising death rates. When per capita resource use is low enough such that birth rates are approximately equal to death rates through time, a dynamic steady-state known as a carrying capacity (*K*) is reached. In this scenario, a population is regulated by the density-dependence of resource availability. It is not, however, clear whether density-dependence is operating in the human population and what the carrying capacity of humanity is at the global scale [16,17].

The effect of population size on population growth rates is mediated through the availability of resources to individuals. Although many resources may influence birth and death rates (e.g., water), energy is a uniquely universal currency because all forms of work require energy expenditure. This applies to the metabolic rates of individuals in wild populations [18] as well as to the industrial energy use of modern human populations, as energy is used to harvest food, deliver water, and provide health care [19–22]. We therefore suggest that an important step towards an integrated, mechanistic understanding of regulation in the global human population is an understanding of how energy supplies are linked to population size.

Generally, the relationship between energy use and population size can be written as a power law: *E*_*tot*_ = *e*_0_ *N* ^*ε*^, where *E*_tot_ is the total energy used by the population, *e*_0_ is a scaling constant, *N* is population size, and ε is a scaling exponent [18,23,24]. The steepness of the scaling relationship, captured by ε, distinguishes three categorically distinct types of growth regimes. First, Malthusian growth occurs when ε < 1 (i.e., sublinear scaling of energy use with population size), as per capita levels of energy use decline with increasing population size, generating the typical negative density dependence that limits population growth. However, for growth regimes characterized by ε ≈ 1 (linear scaling), energy is not limiting and the population may grow exponentially. This is because the per capita levels of energy use are independent of population size, allowing population growth rate to remain approximately constant through time. Third, the super-exponential growth regime is characterized by ε > 1 (super-linear scaling), indicating a positive feedback between population size and the ability of the population to access and use energy. When ε > 1, the population is effectively moving further below carrying capacity through time in spite of the fact that it is increasing in total size. Thus, the hypothesis that the human carrying capacity has increased through time is equivalent to the hypothesis of a super-linear scaling between population size and energy use.

**Fig 1:**
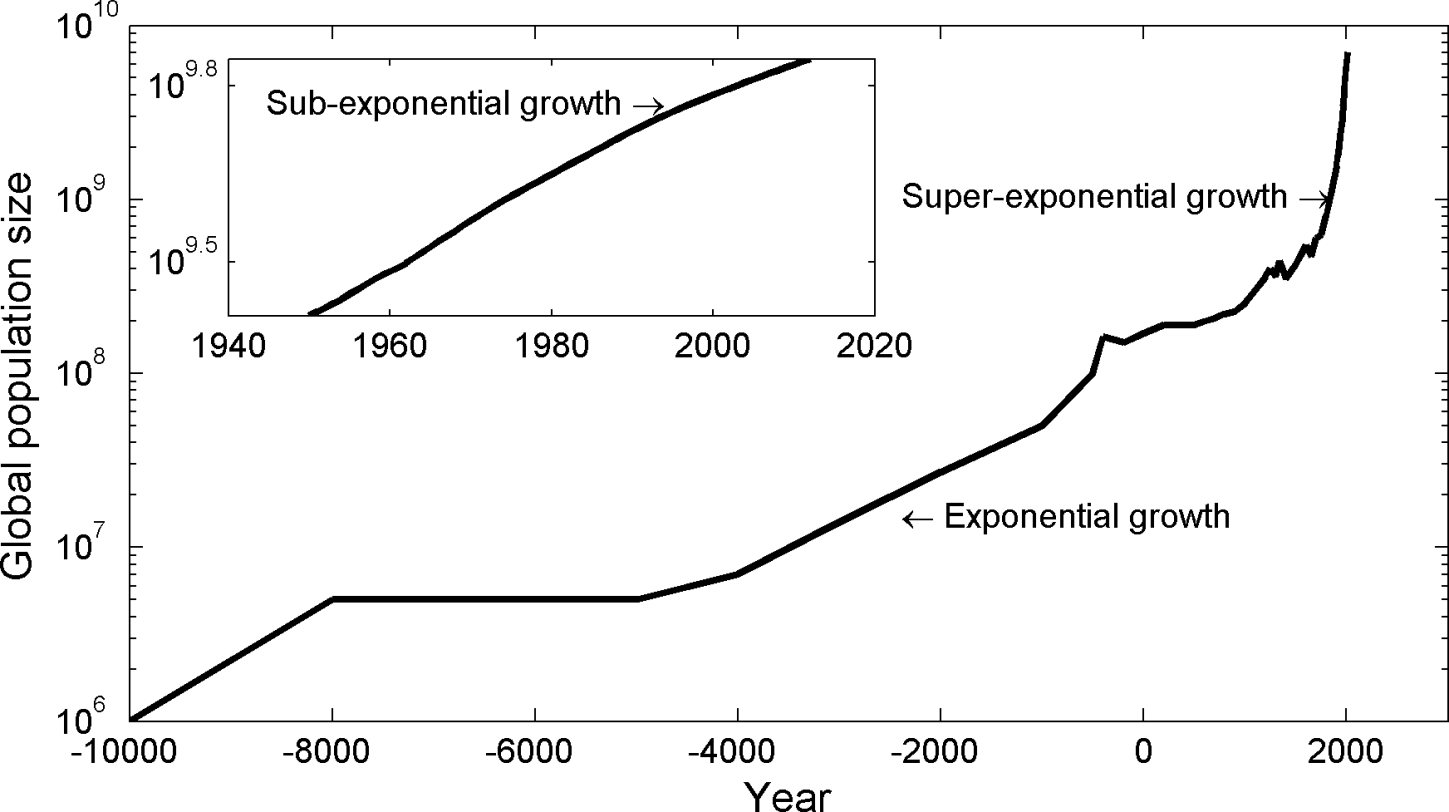
Global population size through time. The inset shows a period of sub-exponential growth in recent history. Data from [25].

Interestingly, the history of global human population growth has included periods characterized by all three growth regimes (density-dependent, exponential, and super-exponential; Fig 1 [25]). For example, super-exponential occurred around the mid-1900s, exponential growth occurred from ~4000 to ~1000 BC, and sub-linear growth has been occurring ~1980 through today (Fig 1, inset). It is well known that throughout this time, global energy use increased with the size of the human population [26], yet it is unclear what the level of energy yield (ε) has been and whether it has varied in time or space. That is, it is only known that ε > 0 on average, but not which of the three regimes have been characteristic at which periods or how the value of ε varies through time. Nonetheless, there is growing support for the idea that the exponential and super-exponential growth seen historically for industrial human populations was enabled by positive feedbacks from population size to carrying capacity [5, 16,27–29]. This feedback could happen in several ways. First, harnessing novel energy sources may free societies from “photosynthetic” energy constraints, as seen in England in the early phases of the Industrial Revolution [30]. Second, information and transportation networks may improve the efficiency of extraction, processing, storage, and transportation of energy [28,31–34]. And finally, an increasing diversity of economic roles could enhance the ability of the population to extract and use resources [35]. In short, there are good reasons to believe that humans are at least episodically capable of having the super-linear energy yields that can cause carrying capacity to increase. Current evidence, however, does not support this hypothesis. A recent cross-country examination suggests ε = 0.75 [36], which would not lead to exponential or super-exponential growth and is therefore inconsistent with the idea that carrying capacity has been increasing through time. We suggest that a within-country analysis of energy use through time is needed for determining whether a positive feedback from population size to energy use exists.

In addition to helping understand the dynamics of the human population, the link between energy use and population size is crucial for developing a comprehensive strategy for global sustainability. Energy use is inextricably linked to population dynamics as well as the manufacture and distribution of goods and services that support food production and our socio-economic system overall [21,37,38]. Yet many question the ability of humans to keep extracting energy at the same rate as we have in the past and suggest that resource constraints may occur at the national or global level in the future [21],[39–42]. It is unknown how changes in resource supply would alter ε and change the growth regime. In addition, instability in the yield of energy, measured as variance in ε through time, could be linked to social or political processes that could in turn influence the population’s growth regime.

Here we investigate the scaling of energy use with population size through time, for the globe and for countries with long-term records on population size and energy use. We evaluate whether the scaling of energy use with population size is quantitatively in agreement with the hypothesis that exponential and super-exponential population growth in humans have been enabled by energy availability keeping up with or outpacing population size, respectively. We further investigate temporal variability in the yield of energy in light of historical factors.

## Materials and Methods

Total primary energy use (also known as energy consumption, primary energy consumption, or total primary energy supply, but hereafter just energy use) reflects the ability of human populations to extract and use a variety of energy types from the environment. Typically, energy use for a given country in a given year is the simple sum of all the energy actually used that was derived from oil, coal, wind, and all other sources. It includes all within country production and imports, and excludes losses to heat and exports, meaning that the heat waste from a power plant or electricity loss from the grid is not counted toward the energy use [22,42]. We used long-term data on total energy use and population size for Sweden (from 1800 to 2000 [43]) and England and Wales (from 1560 to 2000 [44]). We paired long-term data on energy use in the United States [45,46] with population size data from the US Census Bureau [47–49]. Data for the United States covered 1790 to 2012, during which time the United States did not have a constant geographic area. We include all the data for the United States for completeness, but the data are most insensitive to area effects after about the mid-1800s. For the world, we combined population and energy data from several sources [42,50,51].

**Fig 2:**
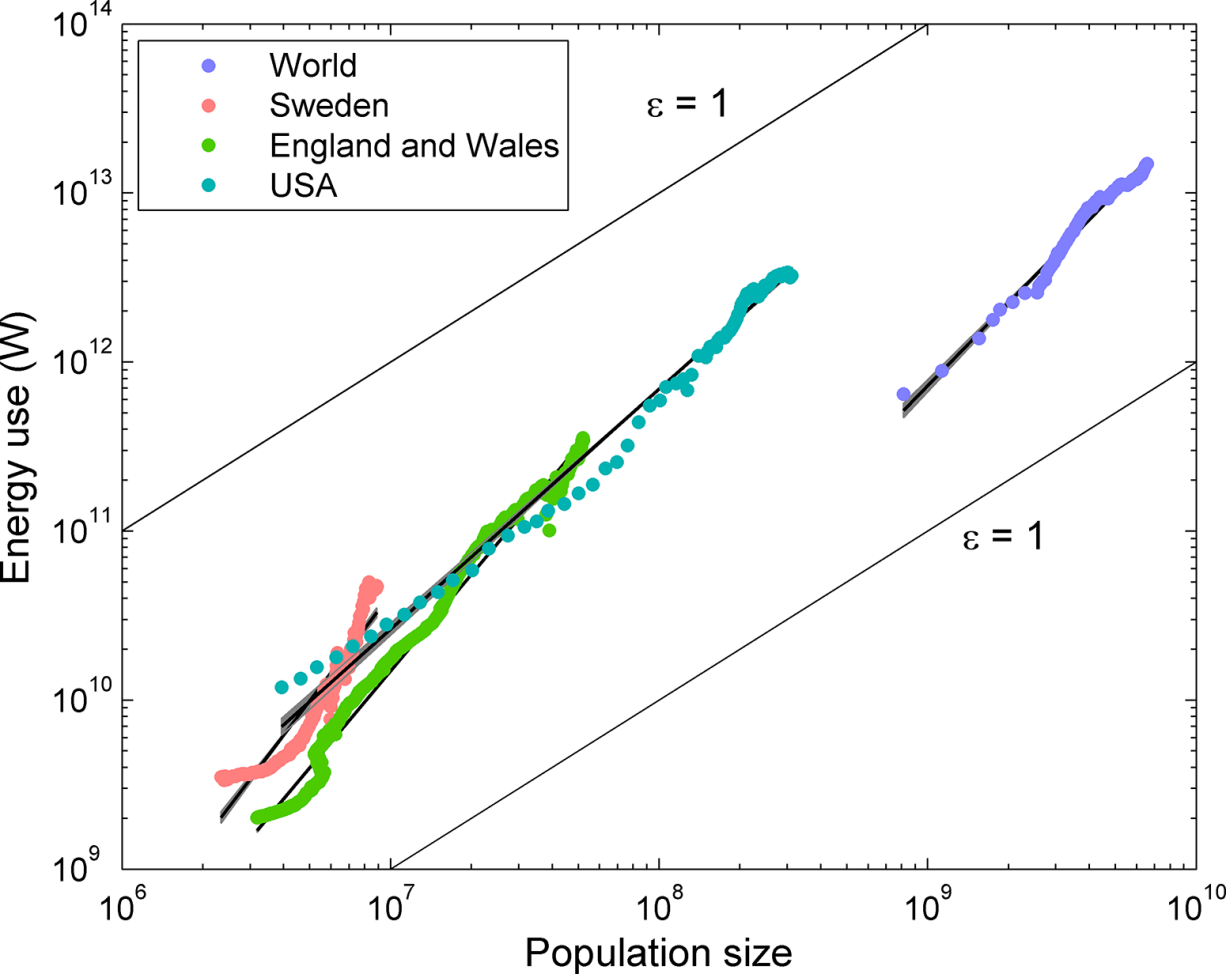
Relationship between energy use (W) and population size for the world, the United States, Sweden, and England and Wales through time. The relationships are highly variable, but overall, the slopes are greater than one (that is, the exponent in the power-law function relating energy use to population size overall), indicating support for a positive feedback between population size and energy use. Lines with slopes of one (ε = 1) are shown as reference. The black lines show overall fits and gray shaded regions show 95% confidence intervals on the regression lines.

For all four data sets, we analyzed the relationship between energy use and population size using ordinary least squares regression. We assessed the slope of the relationship between the natural log of energy use on the natural log of population size (ε) using moving windows of 19 consecutive measurements through time. This procedure accommodated the considerable non-linearity of the relationship in log space (Fig 2) and also provided an objective means of identifying changes in slope and variability through time. These moving windows were then smoothed over 20 consecutive measurements. Other choices for the size of the moving regression and smoothing window produced qualitatively the same results, but shorter windows tended to exaggerate the variability, due to over-fitting, and longer windows were insensitive to the non-linearities. We also tested for a difference in the overall slope before and after the industrial revolution in England and Wales (1780) and Sweden (1830) using a *t*-test.

## Results

**Fig 3:**
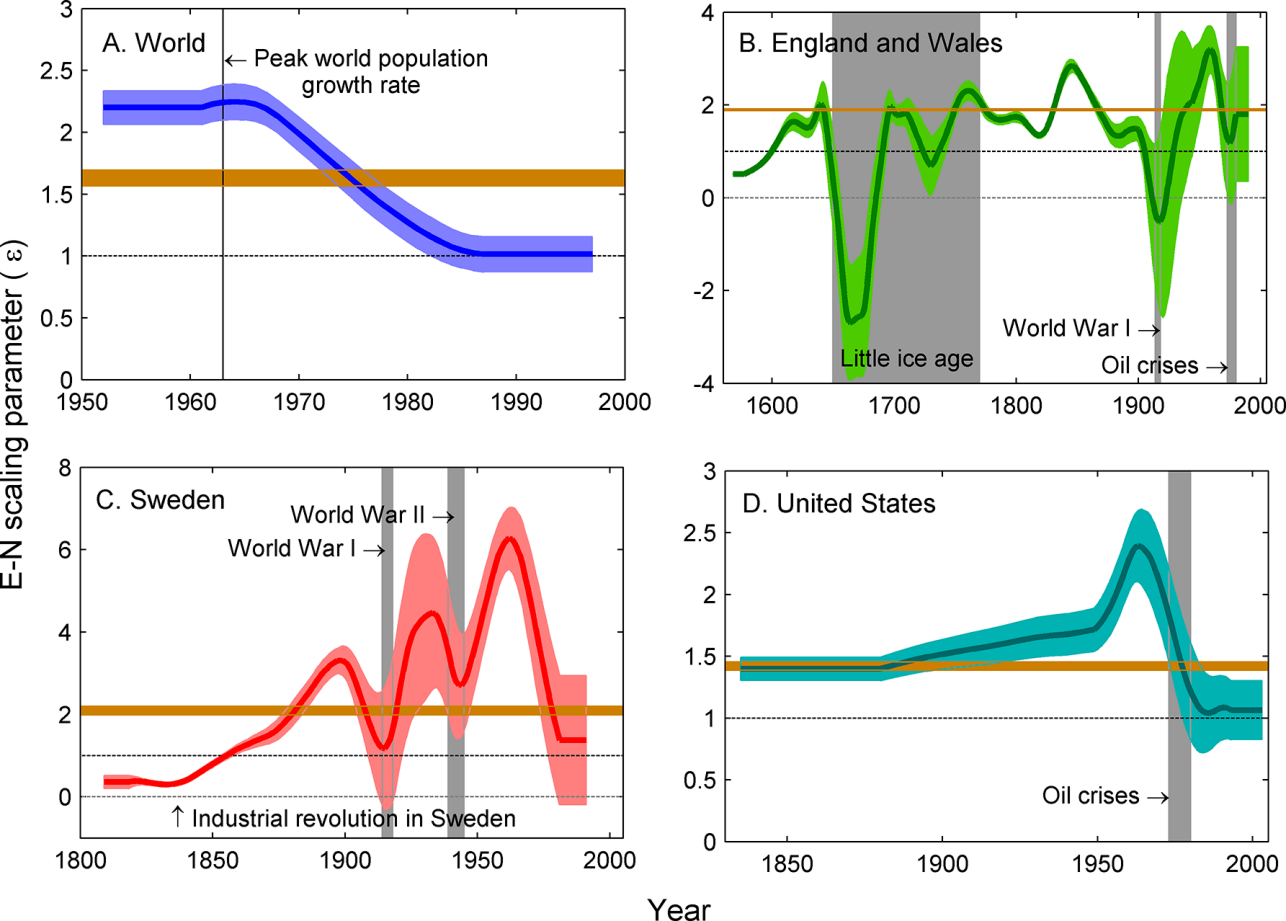
The scaling parameter for ε has been highly variable through time. Each panel shows the running mean of ε (slope of the regression of log*E* on log*N*, see methods) with a 19-year window smoothed over 20 years. The light brown bar shows the confidence range of mean slope over the entire time period. **A**. For the world, ε showed a pronounced shift from a little over 2 to 1 from the 1960’s to the 1980’s, with the beginning of this decline coinciding with the peak world population growth rate in 1963 [9]. **B**. For England and Wales, ε was highly variable, plummeting during the Little Ice Age and during World War I and the Oil Crises of the 1970s. **C**. Sweden showed an increase in ε after the Industrial Revolution but also showed a decline in ε during both world wars. D. The United States showed a steadily increasing e until about the 1960s when it showed a severe drop coinciding with the Oil Crises of the 1970s.

The scaling exponent relating total energy use to population size (ε; Fig 2) was clearly greater than 1 overall for all four time series. Energy use increased superlinearly with population size for Sweden (ε = 2.09, 95% ci’s = 1.98–2.20, *R* ^2^ = 0.88), England and Wales (ε = 1.90, ci’s = 1.88–1.92, *R* ^2^ = 0.99), the United States (ε = 1.42, ci’s = 1.39–1.45, *R* ^2^ = 0.99), and the world (ε = 1.63, ci’s = 1.57–1.69, *R* ^2^ = 0.98). The time series were non-linear in log space, indicating temporal variation in the yield of energy. The moving window regressions revealed fluctuations that were large enough for ε to drop below 1 and even 0 for some periods of time, indicating density-dependent population regulation, especially for England and Wales (Fig 3A). The standard deviation of ε varied widely through time for the United States, Sweden, and England and Wales (Fig 3B). Strong bursts of variability were temporally associated with World Wars I and II, the oil shocks of 1973–1980, and the Little Ice Age for England and Wales (Fig 3B). The mean ε differed significantly before and after the industrial revolution both for England and Wales (1.07 before, 1.73 after, *t* = -6.35, d.f. = 421, *p* < 0.001) and Sweden (0.35 before, 2.50 after, *t* = -4.27, d.f. = 181, *p* < 0.001).

Variability in the estimates of ε naturally follow shifts in its magnitude (Fig 3). For the three country-level data sets, there were consistent spikes in the standard deviation of ε during major events (e.g., Little Ice Age) and as major events approached (world wars, stock market crash, and oil crises).

## Discussion

Throughout history, growing human populations have used more and more energy [26], yet whether the pace of energy yield with population growth has been fast enough to account for exponential and super-exponential growth has been unknown. Our results support the hypothesis that carrying capacity has increased faster than population size through time [5], as the yields of energy that would be needed to support this increase have occurred at the country level (albeit for a few early industrialized countries) and the world as a whole (Fig 2). Increasing per capita energy yields have in essence alleviated the effects of density-dependence for these countries. Although direct observations of increases in carrying capacity have not been made [16], the super-linear yield of energy will have allowed each new person, on average, access to more energy than the one before. This positive feedback played a necessary role in allowing the global population to continue growing very rapidly.

A high value of ε may be related to technologies that have drastically improved the capacity for humans to extract energy from the environment, efficiencies in the structural organization of societies, and a diversification of economic roles [28,32,35]. Undoubtedly, the increasing use of non-renewable resources has maintained ε > 1 for most of the last 150 years [30]. It is likely that the industrial revolution spurred an increase in the value of ε, as overall the time-averaged slopes were significantly higher after the industrial revolution than before it for both England and Wales and Sweden. Nonetheless, some periods of super-linear scaling of energy use with population size occurred for a century prior to the Industrial Revolution, at least in England and Wales, indicating that it is not just industrialization that has helped keep energy yields high through time (Fig 3B).

The pre-Industrial Revolution energy yields were approximately linear for England and Wales but were sublinear for Sweden. This difference suggests a qualitatively different population dynamic in the two countries before the Industrial Revolution began. One possible explanation for the difference is in the speed at which the Industrial Revolution began in the two countries. Although there is debate, the consensus view is that the time at which the Industrial Revolution took hold in England was around 1760–1780, and this is based on particularly visible signs of economic growth, like increases in foreign trade, and less so on the development of extractive technologies that reduced the Malthusian constraints of labor and land [52–54]. Indeed, coal did not become a major part of the energy use in Sweden until the end of the 19^th^ Century, with firewood and human muscle carrying most of the energy burden until post-1900 [43]. This delayed shift to fossil fuel reliance may underlie the later increase in exponent for Sweden as compared to England. Likewise, the technologies that made industrial economies possible were developed gradually in England, which could have kept the value of ε closer to 1 for some time before the Industrial Revolution really began to have a dramatic impact on economic productivity. In contrast, if extractive technologies were adopted more rapidly and as a package, rather than piecemeal through time, then this could delay the start of the Industrial Revolution but increase its pace, causing the value of ε to shift more rapidly from sublinear to super-linear (see Fig 3C).

**Fig 4:**
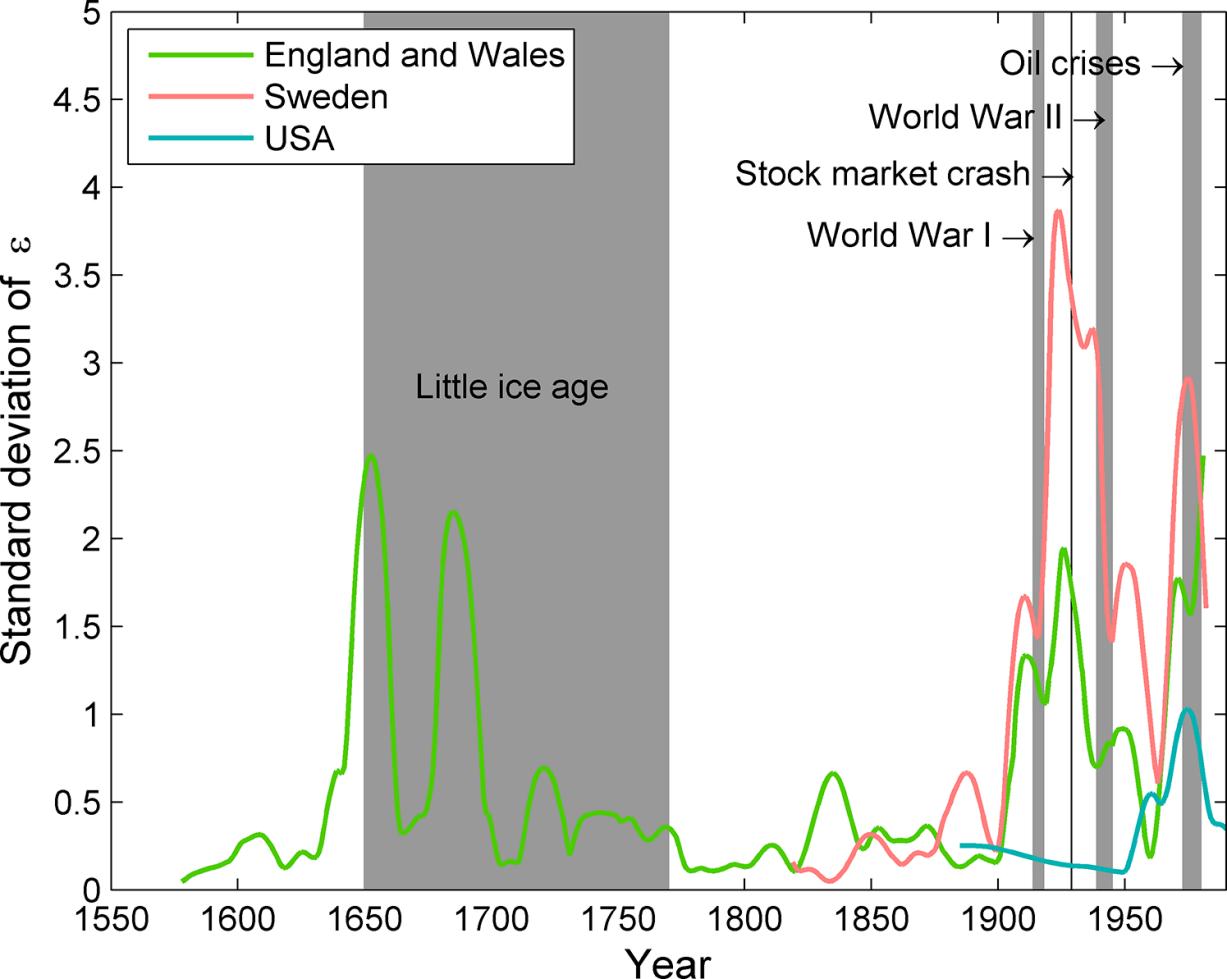
Variation in the scaling parameter e increased as major socio-political events approached and during the Little Ice Age for England and Wales. The world data set is not long enough to include in this analysis.

Despite the overall super-linear scaling, the yield of energy with population size was highly variable through time (Figs 3 and 4). At the global level, an earlier superlinear yield (ε > 1) has gradually shifted down to an approximately linear yield. England and Wales showed several periods of sublinear (ε < 1) and even negative scaling (ε < 0), indicating that even industrialized nations may occasionally experience per capita levels of energy supply that decline as the population grows. This pattern suggests that the increasing demands of the population occasionally do push up against the upper limits of population’s capacity to acquire and use energy. Although not arising to the same degree in each country, there were notable decreases in the scaling exponents during both world wars and the oil crises of the 1970s.

Notable increases in the variability of the exponent ε occurred during three periods (Fig 4). First, England and Wales showed increased variability in the energy yield during the Little Ice Age. Second, European nations showed high variability across the World Wars. Finally, increased variance was associated with oil shocks of the 1970s. At this point we note only the temporal correlations between these political and environmental crises and increased variation in the relationship between energy and population, rather than inferring causation. It is just as possible that political upheaval altered the ability of societies to extract resources as it is that a change in resource availability influenced political stability. Although our results show a clear link between socio-economic stability and population dynamics [55], the world as a whole displayed less variability overall than countries, suggesting that there is some capacity of many countries at a larger scale to buffer local variability [1].

It is likely that a continuation of a positive yield of energy with population size depends strongly on the replacement of fossil fuel sources with renewable energy sources. It is not clear at this time whether these replacement fuel sources could maintain ε where it has been or whether it will drop, bringing the global population back to a density-dependent, Malthusian growth regime. Some have argued that economic or physical limits will eventually slow the supply of energy to humanity’s global socio-economic system [21]. Furthermore, the increased use of energy may have negative indirect consequences through larger amounts of waste produced and larger ecological footprints [56,57]. Nonetheless, more distributed energy resources less subject to political and social upheaval could reduce the variance in ε. In light of the theory that increasing variability is an early-warning signal of state changes [58], greater effort to stabilize the yield of energy may be warranted.

Although our results support the notion that rapid increases in resource availability helped generate exponential and super-exponential population growth in humans, reductions in fertility as energy use and wealth have increased (the demographic transition) suggest that the role of resources in population regulation could be operating by an entirely different pathway [9,17,22,59]. Demographers are far from achieving a widely accepted explanation for causes of fertility reduction [9,60], but energy use is clearly linked to a broad suite of life history traits related to fertility and mortality [22]. Recent research shows that economic factors are related to fertility, but in regression models, indices of cultural transmission of fertility norms are often significant as well [61,62]. It may be that resources play a more direct role than conventional wisdom allows, even if the mechanism is different when individuals can use energy at rates several-fold above their physiologically-determined metabolic rates [22]. Recent evidence of fertility increases in countries with access to high levels of energy, however, point to serious deficiencies in our understanding of how resource use, or development in general, and birth rates are linked [63].

Although greater efficiency in the use or generation of energy may obviate the need for high energy yields, our results highlight the problem of what would keep ε near or greater than one in the future. New technologies must be developed quickly enough to keep pace with population size and the concurrent energy demands, or alternatively, societies could find ways to reach higher mean standards of living with less energy per capita. The historical energy yields demonstrated here show that each new unit of population requires more energy than the one before it, a regime of growth that places great demands on innovation while generating high per capita environmental costs [28,31,40,64]. There is a dynamic tension between harnessing creativity to keep the rate of technological development fast enough to outpace Malthusian limits and maintain population size, stability, and quality of life such that innovations can continue to have positive feedbacks with population size [65]. There is no empirical or theoretical guarantee that innovations and technology can keep pace with population size, yet many assume that the rapid growth since the Industrial Revolution is evidence that the growth observed today and in recent history is necessarily self-sustaining in the long run [66,67]. Put simply, super-linear energy yields are the primary reason for the impression that growth is self-sustaining or that ecological carrying capacities are not applicable to human societies in the modern era.

## Conclusions

The finding that super-linear energy yields typify the growth of the modern human population is an important additional piece of an emerging picture about how energy structures human societies at multiple levels of organization. Historical demographers like Wrigley [30] and Warde [44] revealed the importance of energy use as a driver for economic growth and emphasized how land and labor constraints are released with the transition to fossil fuels. Similarly, but from a different analytical point of view, macro-ecological studies have revealed strong associations between energy use and the drivers of population growth [6,21,67,68,69], energy use and economic activity [21], population size and economic activity [28], and population size and structural organization in cities [70]. Some classical anthropological research has stressed the fundamental role of energy on societal processes [71,72] and economists have linked oil prices and availability to economic growth [73]. Finally, work on ‘social metabolism’ [38] has linked material flows to transportation [31], efficiency and affluence [65], and ecological impacts [74]. The interrelatedness of all these processes suggests that the dynamics of the human population revolve around a complex mixture of energy-mediated ecological, evolutionary, economic, political, social, and technological processes. A more synthetic, interdisciplinary, and mechanistic view of human population dynamics is urgently needed, as the size of the human population is a central component of a sustainable future.
